# Using machine learning to predict adverse events in acute coronary syndrome: A retrospective study

**DOI:** 10.1002/clc.24127

**Published:** 2023-08-31

**Authors:** Long Song, Yuan Li, Shanshan Nie, Zeying Feng, Yaxin Liu, Fangfang Ding, Liying Gong, Liming Liu, Guoping Yang

**Affiliations:** ^1^ Department of Cardiovascular Surgery The Second Xiangya Hospital Central South University Changsha Hunan China; ^2^ Xiangya School of Pharmaceutical Sciences Central South University Changsha Hunan China; ^3^ Center of Clinical Pharmacology, The Third Xiangya Hospital Central South University Changsha Hunan China; ^4^ Department of Intensive Care Unit The Third Xiangya Hospital Central South University Changsha Hunan China

**Keywords:** acute coronary syndrome, AKI, all‐cause mortality, machine learning, prognosis

## Abstract

**Background:**

Up to 30% of patients with acute coronary syndrome (ACS) die from adverse events, mainly renal failure and myocardial infarction (MI). Accurate prediction of adverse events is therefore essential to improve patient prognosis.

**Hypothesis:**

Machine learning (ML) methods can accurately identify risk factors and predict adverse events.

**Methods:**

A total of 5240 patients diagnosed with ACS who underwent PCI were enrolled and followed for 1 year. Support vector machine, extreme gradient boosting, adaptive boosting, K‐nearest neighbors, random forest, decision tree, categorical boosting, and linear discriminant analysis (LDA) were developed with 10‐fold cross‐validation to predict acute kidney injury (AKI), MI during hospitalization, and all‐cause mortality within 1 year. Features with mean Shapley Additive exPlanations score >0.1 were screened by XGBoost method as input for model construction. Accuracy, F1 score, area under curve (AUC), and precision/recall curve were used to evaluate the performance of the models.

**Results:**

Overall, 2.6% of patients died within 1 year, 4.2% had AKI, and 4.7% had MI during hospitalization. The LDA model was superior to the other seven ML models, with an AUC of 0.83, F1 score of 0.90, accuracy of 0.85, recall of 0.85, specificity of 0.68, and precision of 0.99 in predicting all‐cause mortality. For AKI and MI, the LDA model also showed good discriminating capacity with an AUC of 0.74.

**Conclusion:**

The LDA model, using easily accessible variables from in‐hospital patients, showed the potential to effectively predict the risk of adverse events and mortality within 1 year in ACS patients after PCI.

## Graphical abstract

In this retrospective study, eight machine learning (ML) models were built to predict patient outcomes. Features with a mean Shapley Additive exPlanations score >0.1 were screened using the XGBoost method as input for model building. Finally, the linear discriminant analysis model was found to be superior to the other seven ML models.

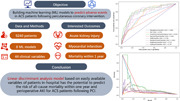

## 1 INTRODUCTION

Acute coronary syndrome (ACS) is a common type of cardiovascular disease and represents one of the leading causes of death worldwide.^1^ Over the past decades, with the advancement of percutaneous coronary intervention (PCI) and other therapeutic strategies, the outcomes have improved among patients. However, up to 30% of patients still suffer from adverse events, which mainly include ischemia, bleeding, kidney failure, and death.^2^ Thus, accurate prediction of adverse events, identification of risk factors, and strengthening the management of high‐risk patients are essential to improve the prognosis of patients.

Massive efforts have been made to construct predictive risk score models that could serve as tools to guide clinical practice and decision‐making. Currently, several influential predictive risk scores, which mainly include Thrombolysis in Myocardial Infarction (TIMI), Global Registry in Acute Coronary Events (GRACE), Patterns of Non‐Adherence to Antiplatelet Regimen in Stented Patients (PARIS), Dual Antiplatelet Therapy (DAPT), and Predicting Bleeding Complications in Patients Undergoing Stent Implantation and Subsequent DAPT (PRECISE‐DAPT), have been used in the clinic. These scores have been developed to predict ischemia, bleeding, and death for patients with ACS or undergoing DAPT after coronary artery stent implantation.

However, the prognoses of ACS patients are influenced by diverse pathological transformations and individual variations,^3^ rendering the accuracy of these scores insufficient for personalized patient management strategies amidst the advancement of precision medicine. Furthermore, the drawback of underestimating or overestimating risks in patients with dissimilar baseline characteristics must not be overlooked. Moreover, most risk prediction algorithms were initially developed for unique ethnicities and may not be suitable for other populations. Based on the traditional statistically derived risk prediction models, the correlation among variables, heterogeneity, nonlinearity, and overfitting also restricted the application, especially in multifaceted data sets with large numbers of features.^4^

Machine learning (ML) methods can overcome the shortcomings of current prediction risk models. As an important branch of artificial intelligence, the advantage of ML is using computer algorithms to identify characteristics in large data sets with numerous, multidimensional, and nonlinear relationships among clinical features to predict various outcomes.^5^ Thus, ML has become a promising adjunct to prevention, diagnosis, treatment, and clinical decision support. A representative ML‐based prediction model is Predicting with Artificial Intelligence Risk after Acute Coronary Syndromes (PRAISE).^6^ This model showed high accuracy in detecting the risk of all‐cause mortality, recurrent acute MI, and major bleeding in ACS patients within 1 year after discharge. However, the study mainly included European samples with little to no inclusion of Asian and African individuals. Some procedure‐related variables and angiographical features, such as the number of diseased vessels, were not included in the model, which may influence the outcome of patients.

The aim of this study was to develop an ML‐based risk stratification model integrating demographics, concomitant drugs and diseases to predict perioperative MI, acute kidney injury (AKI) during hospitalization, and all‐cause mortality within 1 year in patients with ACS undergoing PCI.

## 2 MATERIALS AND METHODS

### 2.1 Data sets

**Figure 1:**
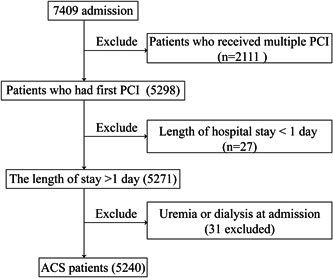
Flow chart. ACS, acute coronary syndrome; PCI, percutaneous coronary intervention.

As shown in Figure 1, a total of 7409 patients undergoing coronary angiography and successful PCI who were hospitalized at the Department of Cardiology of the Third Xiangya Hospital from June 2007 to June 2021 were followed for 1 year. The exclusion criteria were (a) patients who underwent multiple surgeries (*n* = 2111); (b) patients with a hospital stay of less than 1 day (*n* = 27); and (c) patients who presented with dialysis (*n* = 31). In all, 5240 patients were included in the data sets.

The study was conducted in accordance with the Declaration of Helsinki's ethical guidelines and approved by the Medical Ethics Committee of the Third Xiangya Hospital (Ethics approval number: R18030). Considering the minimal risk, the requirement for informed consent was waived for this study.

### 2.2 Study outcomes

All‐cause mortality within 1 year was determined according to the 10th Revision of International Classification of Disease (ICD‐10). MI associated with PCI was defined by an elevation of cardiac troponin (cTn) values >5 times the 99th percentile URL in patients with normal baseline values within 48 hours after PCI. In addition, the evidence should include new pathological Q waves, imaging evidence, or procedure‐related complications.^7^ AKI was considered an absolute increase in serum creatinine level of 0.3 mg/dL within 48 hours or a relative increase of 1.5 times baseline within the prior 7 days according to the guidelines of the Kidney Disease Improving Global Outcomes.^8^

### 2.3 Data processing and feature selection

All data included in the study were extracted from the electronic medical record (EMR) system using SQL. For data processing, the missing values, which were less than 5%, were replaced with the mean value for continuous variables. Moreover, the associated myocardial enzymes were discarded because they included over 50% missing values. All continuous variables were z‐standardized, and categorical variables were one‐hot encoded. To address the problem of imbalanced classes, the SMOTEENN algorithm was applied to process the original data.

Forty‐four potential features that had an impact on ACS were selected based on extensive literature: sex, age, current smokers, BMI, diabetes, hypertension, hyperlipidemia, previous PCI, chronic kidney disease, AKI, diagnosis, numbers of diseased vessels (NDV), Killip class, β‐blockers, angiotensin converting enzyme inhibitors, angiotensin receptor blockers (ARB), calcium channel blocker, morphine, diuretics, P2Y12 inhibitor, hemoglobin (Hb), white blood cell (WBC), red blood cell (RBC), platelet count (PLT), mean platelet volume (MPV), platelet distribution width (PDW), platelet large cell ratio (P_LCR), platelet to lymphocyte ratio (PLR), neutrophil to lymphocyte ratio (NLR), neutrophil (Neu), lymphocyte (Lym), high‐density lipoprotein cholesterol (HDL‐C), low‐density lipoprotein cholesterol (LDL‐C), triglyceride (TG), total cholesterol (TC), troponin, brain natriuretic peptide (BNP), glucose, heart rate, systolic blood pressure, diastolic blood pressure, mean blood pressure, uric acid, ST‐segment elevation MI, and unstable angina/non‐ST‐segment elevation MI. The importance rank of the features was derived from the Shapley Additive exPlanations (SHAP) mean score by the XGBoost method, and a feature importance score >0.1 was subsequently selected as an input in model building. In addition, the SHAP approach was used to explain the effects of all feature contributions on the outcome of each patient.^9^

### 2.4 Model development and validation

After the selection of important variables, the derivation cohort was randomly split into two data sets: a training (70%) cohort and an internal validation (30%) cohort. Eight common ML classifiers, support vector machine (SVM), extreme gradient boosting (XGBoost), adaptive boosting (AdaBoost), K‐nearest neighbors (KNN), random forest (RF), decision tree, categorical boosting (CatBoost), and linear discriminant analysis (LDA), were used to predict adverse events. A ten‐fold cross‐validation method was used to train the data sets in all models. Receiver operating characteristic (ROC) curves along with the area under the curve (AUC), accuracy, recall, specificity, precision, and F1 score were used to evaluate the performance of the models. A specific formula was calculated according to the confusion matrix (true positive, true negative, false positive, false negative).

Accuracy=(TP+TN)/(TP+FP+TN+FN)

Recall=TP/(TP+FN)

Specificity=TN/(TN+FP)

Precision=TP/(TP+FP)

F1score=2×precision×recall/(precision+recall)

Then, the precision/recall curve was used to evaluate binary decision problems on imbalanced data sets.^10^

### 2.5 Statistical analysis

Continuous variables are displayed as the mean and standard deviation, and categorial variables are displayed as percentages or numbers. The *t*‐test or Mann−Whitney *U* test was used for continuous variables when appropriate, and the *χ* ^2^ test or Fisher's exact test was used for categorical variables. A two‐tailed *p* < .05 was considered statistically significant. SPSS 25.0 and Python 3.6 were used for the study.

## 3 RESULTS

### 3.1 Baseline characteristics

As mentioned before, 5240 patients were included in the study. Table 1 shows the characteristics of all cohorts. Overall, the average age of all cohorts was 63.1 years, with 72.8% male. A total of 61.4% and 36% of patients had hypertension and diabetes, respectively. A total of 2.6% of patients died within 1 year, 4.2% had AKI, and 4.7% had MI during hospitalization (the characteristics of AKI and MI cohorts are shown in Supporting Information: Table S1). Age was higher in the death, AKI, and MI cohorts than in each of the corresponding cohorts. Specifically, except for the use of ARBs, diabetes, smokers, lipid‐related indicators, P‐LCR, PDW, MPV, platelets, and TC, other clinical variables showed a statistically significant difference between the nondeath and death groups.

**Table 1:** Baseline characteristics of all patients according to all‐cause mortality.

Characteristics	All (*N* = 5240)	All‐cause mortality		
		Non‐death (*N* = 5102)	Death (*N* = 138)	*p* Value
Age (years)	63.07 ± 10.68	62.94 ± 10.66	69.33 ± 9.86	<.001
Body mass index (kg/m^2^)	24.42 ± 3.03	24.43 ± 3.03	23.84 ± 2.87	.025
Male (%)	3817 (72.8)	3729 (73.1)	88 (63.8)	.015
Smokers				.146
Never	3164 (60.4)	3070 (60.2)	94 (68.1)	
Quit smoking	354 (6.8)	345 (6.8)	9 (6.5)	
Current smoking	1722 (32.9)	1687 (33.1)	35 (25.4)	
Diastolic blood pressure (mmHg)	75.38 ± 11.97	75.45 ± 11.91	72.95 ± 13.61	.015
Systolic blood pressure (mmHg)	126.89 ± 20.53	127.01 ± 20.45	122.57 ± 22.98	.012
Mean arterial pressure (mmHg)	92.55 ± 13.52	92.64 ± 13.45	89.49 ± 15.44	.007
Heart rate (bpm)	75.15 ± 13.83	75.00 ± 13.62	80.72 ± 19.50	<.001
Previous PCI	503 (9.6)	483 (9.5)	20 (14.5)	.048
Numbers of diseased vessels				<.001
1	659 (12.6)	646 (12.7)	13 (9.4)	
2	1113 (21.2)	1087 (21.3)	26 (18.8)	
3	2490 (47.5)	2400 (47.1)	90 (65.2)	
Killip				<.001
1	1340 (25.6)	1318 (25.8)	22 (15.9)	
2	744 (14.2)	716 (14.0)	28 (20.3)	
3	368 (7.0)	346 (6.9)	22 (15.9)	
4	218 (4.2)	196 (3.8)	22 (15.9)	
Diagnosis				<.001
STEMI	1232 (23.5)	1182 (23.2)	50 (36.2)	<.001
NSTE‐ACS	4008 (76.5)	3920 (76.8)	88 (63.8)	
Uric acid (μmol/L)	352.64 ± 104.20	350.92 ± 102.53	416.14 ± 139.85	<.001
Triglyceride (mmol/L)	1.84 ± 1.49	1.85 ± 1.50	1.52 ± 1.13	<.001
Total cholesterol (mmol/L)	4.40 ± 1.12	4.40 ± 1.12	4.25 ± 1.15	.088
Platelets (10^9^/L)	206.06 ± 62.74	205.79 ± 62.31	216.21 ± 76.58	.296
Mean platelet volume (fL)	10.69 ± 1.28	10.69 ± 1.28	10.58 ± 1.43	.316
Platelet distribution width (fL)	15.46 ± 1.91	15.46 ± 1.92	15.52 ± 1.78	.282
Platelet large cell ratio (%)	32.58 ± 7.78	32.60 ± 7.75	31.99 ± 8.59	.231
Lymphocyte (10^9^/L)	1.58 ± 0.64	1.59 ± 0.64	1.36 ± 0.58	<.001
Neutrophil (10^9^/L)	6.07 ± 3.26	6.02 ± 3.21	7.85 ± 4.52	<.001
Glucose (mmol/L)	6.63 ± 2.79	6.60 ± 2.76	7.66 ± 3.76	<.001
HDL‐C (mmol/L)	1.10 ± 0.25	1.10 ± 0.25	1.11 ± 0.26	.532
LDL‐C (mmol/L)	2.36 ± 0.84	2.36 ± 0.84	2.24 ± 0.86	.057
Hemoglobin (g/L)	132.27 ± 17.40	132.52 ± 17.16	122.96 ± 22.90	<.001
WBC (10^9^/L)	8.31 ± 3.27	8.26 ± 3.21	9.90 ± 4.52	<.001
RBC (10^12^/L)	4.33 ± 0.57	4.34 ± 0.56	4.04 ± 0.71	<.001
PLR (%)	151.89 ± 84.89	150.92 ± 84.15	187.50 ± 102.62	<.001
NLR (%)	4.87 ± 4.78	4.79 ± 4.66	7.63 ± 7.33	<.001
BNP (pg/mL)	1789.99 ± 2409.87	1711.00 ± 2274.45	4710.41 ± 4543.62	<.001
Troponin (ng/Ml)	3.53 ± 5.33	3.49 ± 5.31	4.93 ± 5.96	<.001
CKD	207 (4.0)	186 (3.6)	21 (15.2)	<.001
Hypertension	3215 (61.4)	3124 (61.2)	91 (65.9)	.262
Hyperlipidemia	662 (12.6)	652 (12.8)	10 (7.2)	.054
Diabetes	1885 (36.0)	1826 (35.8)	59 (42.8)	.093
ACEI	3252 (62.1)	3188 (62.5)	64 (46.4)	<.001
ARB	1280 (24.4)	1246 (24.4)	34 (24.6)	.954
β‐blockers	4476 (85.4)	4374 (85.7)	102 (73.9)	<.001
Diuretics	1397 (26.7)	1307 (25.6)	90 (65.2)	<.001
CCB	1683 (32.1)	1643 (32.2)	40 (28.9)	.424
Ticagrelor	1478 (28.2)	1433 (28.0)	45 (32.6)	.244
Morphine	857 (16.4)	805 (15.8)	52 (37.7)	<.001

*Note*: Continuous variables are displayed as mean ± standard deviation, while categorical variables are as numbers (percentage %).

Abbreviations: ACEI, angiotensin converting enzyme inhibitors; ACS, acute coronary syndrome; ARB, angiotensin receptor blockers; BNP, brain natriuretic peptide; CCB, calcium channel blocker; CKD, chronic kidney injury; HDL, high‐density lipoprotein; LDL, low‐density lipoprotein; NLR, neutrophil to lymphocyte ratio; NSTEMI‐ACS, unstable angina/non‐ST‐segment elevation myocardial infarction; PCI, percutaneous coronary intervention; PLR, platelet to lymphocyte ratio; RBC, red blood cell; STEMI, ST‐segment elevation myocardial infarction; WBC, white blood cell.

### 3.2 The selected features and ML models

**Figure 2:**
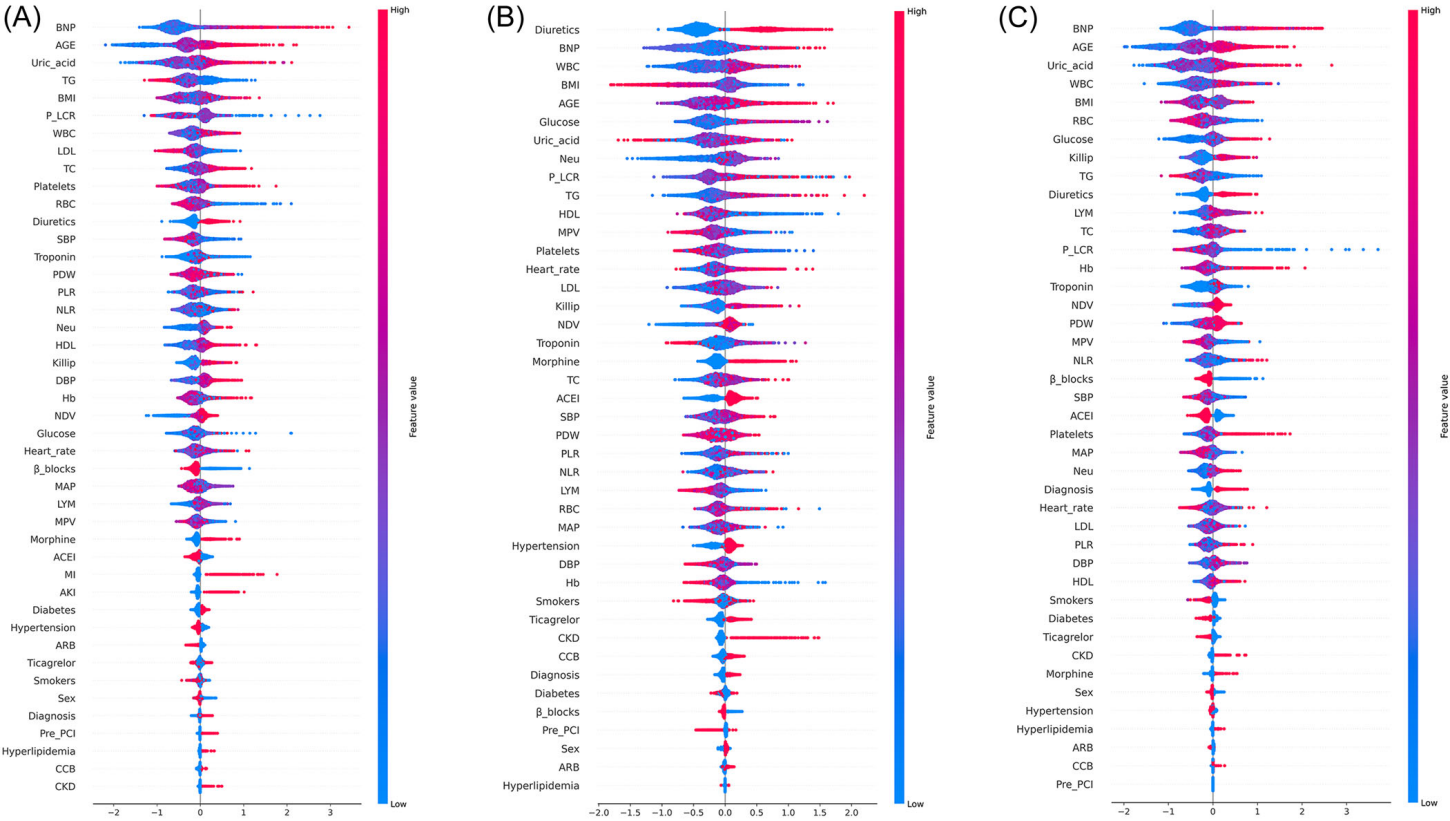
SHAP summary plot of the features by using the XGBoost model. A dot represents a patient and is created for each feature attribution value for the model. Dots are colored according to the values of features for the respective patient and accumulate vertically to depict density. The dot color is redder as the feature value increases and bluer as the feature value decreases. For the adverse event outcome, a higher SHAP value of a feature reflects a higher probability. If the SHAP value of a feature increases in the same direction as the *x*‐axis, this indicates that an increase in the value of the feature increases the incidence of adverse events. Conversely, it indicates that an increase in the value of the feature decreases the incidence of adverse events. (A) SHAP value for all‐cause mortality. (B) SHAP value for AKI. (C) SHAP value for MI. AKI, acute kidney injury; MI, myocardial infarction; SHAP, Shapley Additive exPlanations.

The variable importance scores for each outcome according to the permutation importance method in the XGBoost model are shown in Supporting Information: Figure S1. To interpret ML models, SHAP values were used to visualize and explain how these features affect events. Figure 2 summarizes the SHAP value plot by combining feature importance with feature effects. Overall, approximately 30 variables were selected based on the SHAP *p* value >.1 for the outcomes. Herat function (BNP, troponin, Killip, NDV), age, uric acid, and cell blood count (WBC, RBC, Hb, Neu, Lym) were ranked as relatively important features for each outcome prediction. In addition, lipid metabolism (TG, TC, HDL‐C, LDL‐C), glucose, blood pressure, and platelet‐associated parameters (PLT, MPV, PDW, P‐LCR) were also important predictors for all three outcomes. For AKI, morphine was only selected as a potential predictor that reflects the specific underlying pathogenesis of disease development.

### 3.3 Prediction of the ML models

**Figure 3:**
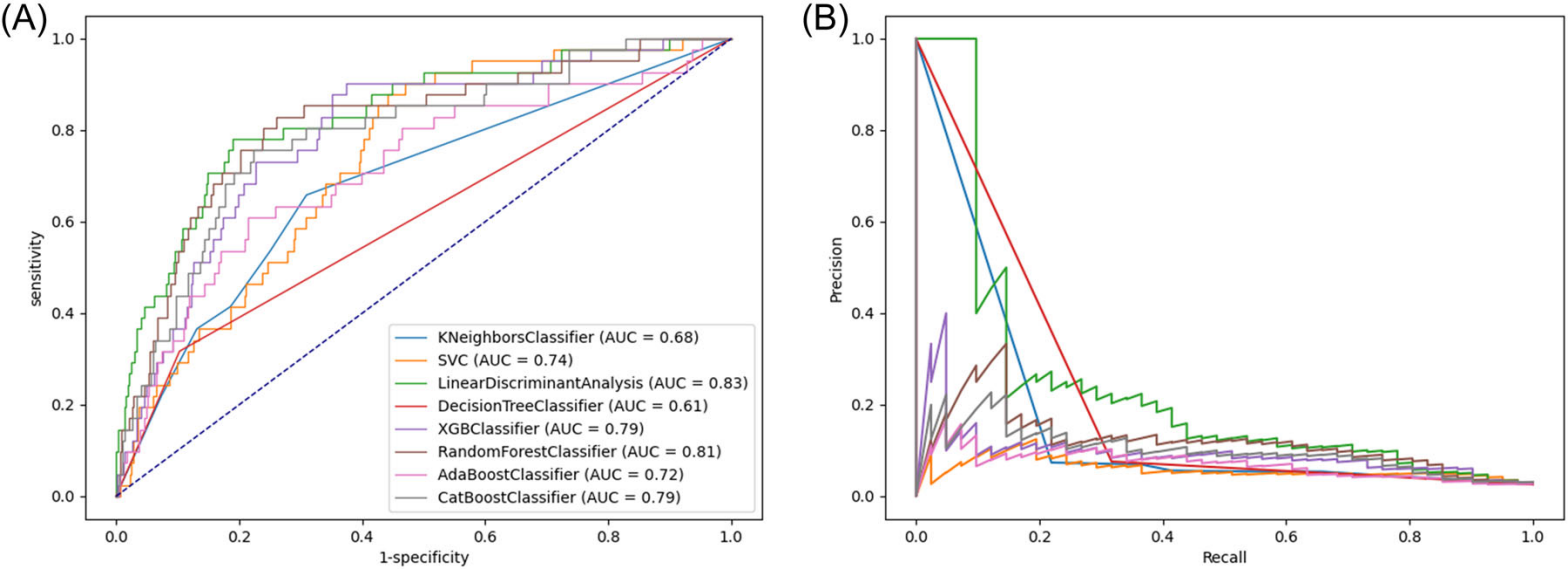
Receiver operating curves and PR curves. In this study, we trained eight models: KNN, SVM, AdaBoost, XGBoost, CatBoost, random forest, decision tree, and LDA. (A) ROC curve; (B) PR curve. KNN, K‐nearest neighbors; LDA, linear discriminant analysis; PR, precision/recall; ROC, receiver operating characteristic; SVM, support vector machine.

**Figure 4:**
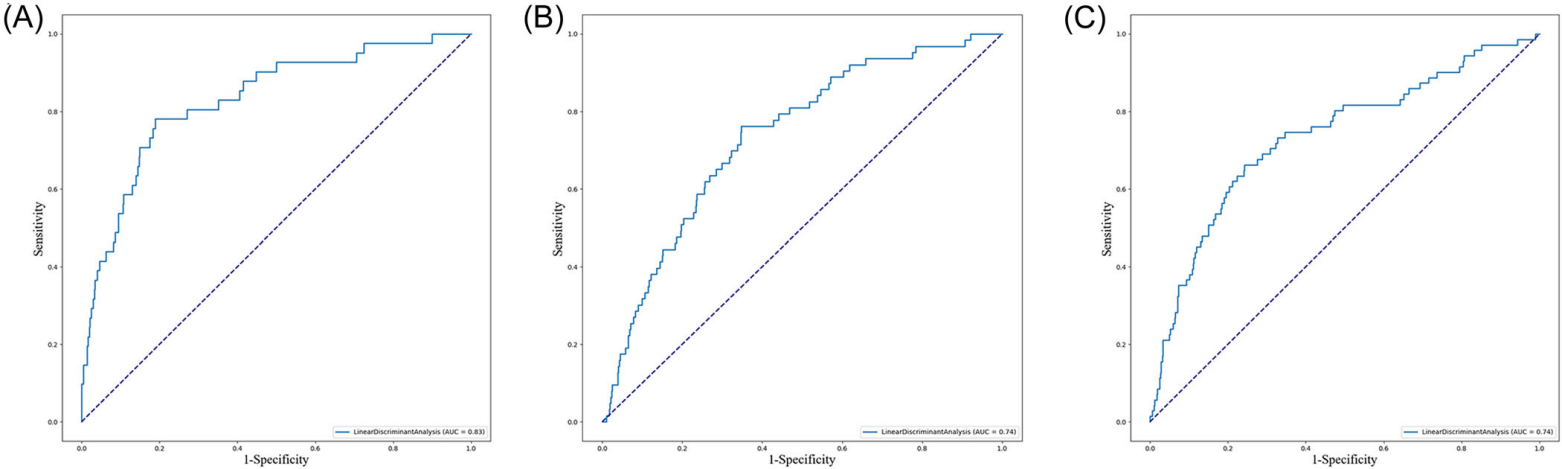
Receiver operating curve of the LDA model for all‐cause mortality, AKI, and MI. (A) All‐cause mortality; (B) AKI; (C) MI. AKI, acute kidney injury; LDA, linear discriminant analysis; MI, myocardial infarction.

For all‐cause mortality, the discriminative performance of the eight ML models is displayed by the ROC curves in Figure 3. Supporting Information: Table S2 presents the confusion matrix and evaluation metrics for all ML models. The LDA model exhibited the best discrimination with an AUC of 0.83, followed by RF (AUC 0.81), XGBoost (AUC 0.79), CatBoost (AUC 0.79), SVM (AUC 0.74), and AdaBoost (AUC 0.72). The decision tree and KNN models performed worst, with AUCs of 0.61 and 0.68, respectively. The range of specificity was 0.2−0.68, and the recall was 0.81−0.96. In general, the LDA model had the best performance among the models when comprehensively evaluating the AUC, accuracy, specificity, recall, precision, and F1 score. Additionally, the PR curves of all models are shown in Figure 3. Figure 4 shows the performance of a single LDA model with an AUC of 0.83 for all‐cause mortality, 0.74 for AKI, and 0.74 for MI (more details in Supporting Information: Table S3). Overall, the LDA model was better than the other models for predicting all‐cause mortality, AKI, and MI in ACS patients.

## 4 DISCUSSION

In this study, we demonstrated that ML models showed good discrimination for the prediction of all‐cause mortality, AKI, and MI in patients with ACS who underwent PCI. Additionally, we identified important predictors of adverse events from EHR. These findings enrich the risk factors for events and have potential future applications in clinical practice. According to the abnormal indicators, early intervention to improve the prognosis of patients is necessary.

Identifying patients at high risk of developing AKI, MI, and poor outcomes remains a challenge in cardiovascular medicine.^11^, ^12^ Although traditional risk factors are helpful to identify high‐risk populations, they are limited for individual risk assessment. Even when using global summary scores, over‐ or undertreatment is inevitable. Thus, accurate prediction of adverse events still represents an unmet need. ML algorithms have achieved good performance when assessing high‐dimensional and nonlinear relations among features.^6^

In contrast to the prior study, we included 44 variables and employed eight machine‐learning models. We demonstrated the predictive power of the models, and the LDA model was superior to the other models, with an average AUC of 0.83. A previous study verified the usefulness of ML techniques in predicting the diagnosis and prognosis of ACS.^13^, ^14^ However, only one ML algorithm was used to predict in‐hospital mortality, 30‐day CHF rehospitalization, and 180‐day cardiovascular death.

Age, BMI, heart function, lipid metabolism, uric acid, glucose, platelet‐associated parameters, and concomitant drugs were considered important variables to predict adverse events. Consistent with a previous study, older patients usually suffered from concomitant comorbidities, such as hypertension, diabetes, dyslipidemia, and kidney disease.^15^ Obesity, manifested as a higher BMI, is considered a risk factor for mortality in the general population. However, an obesity paradox phenomenon, higher BMI with better prognosis, was found in ACS patients.^16^ In our study, BMI was slightly lower in the all‐cause death cohort, and the so‐called “obesity paradox” was still not well understood. Many studies have confirmed that uric acid is an independent risk factor for long‐term all‐cause death in ACS patients.^17^ Moreover, impairment of glucose and lipid metabolism triggers the development of artery plaques and induces the occurrence of adverse events.^18^ Platelets play a vital role in ACS, and their reactivity influences prognosis. Studies have reported that a baseline higher MPV is associated with more cardiovascular events.^19^ A meta‐analysis of eight studies also indicated that higher platelet levels at baseline increased the risk of mortality in ACS patients.^20^ BNP, troponin, Killip class, and multivessel lesions are considered markers of cardiac function. Additionally, these markers have been developed as diagnostic and prognostic tools for ACS.^21^ Perioperative MI and contrast‐induced AKI (CI‐AKI) could lead to markedly high mortality after PCI, and we innovatively included perioperative MI and AKI for predicting all‐cause mortality. However, our model did not reveal them as important confounding variables, and the performance of AUC did not obtain a great improvement. In other words, the adverse effects may be masked by other factors. Additionally, the inflammatory response plays an important role in the progression of atherosclerosis, and PLR and NLR have been the classical markers of inflammation in cardiovascular disease. A previous study demonstrated a higher occurrence of major adverse cardiovascular and cerebrovascular events (MACCEs) following an increased PLR‐NLR.^22^ Therefore, these inflammatory indices were also used as variables to predict prognosis in our study. Several ML models were developed to predict 30‐day mortality, MACCEs, and 1‐year mortality in ACS patients following PCI.^23^, ^24^, ^25^ However, these models were focused on comparing the performance of ML models and traditional logistic regression and GRACE scores. Additionally, they used several variables and one ML algorithm, which may affect the interpretation of the model.

AKI is a common complication among patients undergoing interventional procedures, with a reported incidence of up to 30%.^26^ AKI is directly associated with both a fivefold increase in intrahospital mortality and an increased risk of end‐stage renal failure in the long term.^27^ An updated simple risk score (Mehran 2 CA‐AKI risk score) that included age, clinical presentation, eGFR, congestive heart failure, diabetes, hemoglobin, basal glucose, and left ventricular ejection fraction could accurately discriminate the risk of AKI, and AKI occurrence was strongly associated with 30‐day all‐cause mortality (HR: 14.64, 95% CI: 10.04−21.34).^28^ Hence, this score could be widely utilized in the clinic based on traditional methods. However, the logistic regression model is theory‐driven and requires several putative conditions, such as multiple collinearity problems, a linear relationship between the dependent and logit value of the independent variable, and no outliers. The ML model integrated preoperative variables and intraoperative time‐series physiological data to predict AKI after cardiac surgery, and the performance was superior to that of the traditional logistic regression model (AUC 0.843 vs. 0.806).^29^ Our results revealed additional novel predictors, such as the use of morphine during hospitalization. Morphine is an opioid and has been recommended for the management of acute chest pain in ACS patients.^30^ Evidence has suggested that opioid overdose can lead to AKI due to dehydration, hypotension, rhabdomyolysis, and urinary retention.^31^

In our study, the AUCs of the ML models were more than 0.70, indicating a good discrimination capacity. Along with the popularization of PCI, there has been an increase in clinical data for patients. Hence, the application of the ML method is useful for big data analysis. From a clinical point of view, early assessment of baseline candidate factors on the outcomes could offer targeting of modifiable factors to further improve the prognosis of ACS patients.

There are still some limitations that should be noted in our study. First, this is a retrospective, single‐center study, making it vulnerable to bias. However, these data, which were derived from the EMR system, reflect real‐world clinical practice and have high generalizability. Second, we focused on 1‐year mortality and AKI. Future models could strive to predict the prevention, diagnosis, and therapy of ACS. Third, although as many relevant characteristics were collected as possible, no intraoperative and postoperative variables were gathered in the study, which may augment the performance of the current ML model.

## 5 CONCLUSION

In conclusion, our study demonstrated that an LDA model based on easily available variables of patients in‐hospital has the potential to predict the risk of all‐cause mortality within 1 year and perioperative AKI for ACS patients following PCI.

## AUTHOR CONTRIBUTIONS

Long Song, Yuan Li, Shanshan Nie, Zeying Feng, Liming Liu, and Guoping Yang conceived the study and participated in its design. Yaxin Liu, Fangfang Ding, and Liying Gong contributed to the data collection and code. Long Song, Yuan Li, Shanshan Nie, and Zeying Feng analyzed the data and drafted the manuscript. Liying Gong, Liming Liu, and Guoping Yang reviewed the manuscript. All authors contributed to the revision of the manuscript and read and approved the submitted version.

## CONFLICT OF INTEREST STATEMENT

The authors declare no conflict of interest.

## Supporting information

Supporting information.

Figure S1 Importance ranking of the candidate features. 1 A: Feature importance ranking for all‐cause mortality; 1B: Feature importance ranking for AKI;1 C: Feature importance ranking for MI.

## Data Availability

Deidentified data from this study are available from the corresponding author upon request. The data are not publicly available due to privacy or ethical restrictions.
