# Supplementary material for: Using machine learning to predict adverse events in acute coronary syndrome: A retrospective study
Source: Clin Cardiol. 2023 Aug 31;46(12):1594–602. doi: 10.1002/clc.24127 (PMC10716319; doi:10.1002/clc.24127)
Supplement: Supplementary file 1 — Supporting information. [file CLC-46-1594-s001.docx]

Supplementary Content

**Table S1 Baseline characteristics of all patients according to acute kidney injury and myocardial infarction.**

| Characteristics | All (N = 5240) | AKI | | | MI | | |
| --- | --- | --- | --- | --- | --- | --- | --- |
|  |  | Non-AKI  (N = 5108) | AKI (N = 222) | P value | Non-MI  (N = 4992) | MI (N = 248) | P value |
| Age (y) | 63.07 ± 10.68 | 62.89 ± 10.61 | 67.10 ± 11.55 | < 0.001 | 63.06 ± 10.57 | 63.22 ± 12.71 | 0.014 |
| Body mass index (kg/m^2^) | 24.42 ± 3.03 | 24.46 ± 3.04 | 23.52 ± 2.54 | < 0.001 | 24.43 ± 3.04 | 24.05 ± 2.86 | 0.13 |
| Male (%) | 3817 (72.8) | 3664 (73.0) | 153 (68.9) | 0.205 | 3626 (72.6) | 191 (77.0) | 0.101 |
| Smokers |  |  |  | 0.061 |  |  | 0.141 |
| Never | 3164 (60.4) | 3017 (60.1) | 147 (66.2) |  | 3017 (60.4) | 147 (59.3) |  |
| Quit smoking | 354 (6.8) | 336 (6.7) | 18 (8.1) |  | 344 (6.9) | 10 (4.0) |  |
| Current smoking | 1722 (32.9) | 1665 (33.2) | 57 (25.7) |  | 1631 (32.7) | 91 (36.7) |  |
| Diastolic blood pressure (mmHg) | 75.38 ± 11.97 | 75.47 ± 11.97 | 73.42 ± 11.72 | 0.007 | 75.47 ± 11.94 | 73.56 ± 12.44 | 0.157 |
| Systolic blood pressure (mmHg) | 126.89 ± 20.53 | 126.98 ± 20.52 | 124.89 ± 20.76 | 0.141 | 127.19 ± 20.42 | 120.89 ± 21.95 | 0.297 |
| Mean arterial pressure (mmHg) | 92.55 ± 13.52 | 92.64 ± 13.52 | 90.58 ± 13.41 | 0.027 | 92.71 ± 13.46 | 89.34 ± 14.34 | 0.243 |
| Heart rate (bpm) | 75.15 ± 13.83 | 74.90 ± 13.54 | 80.89 ± 18.49 | < 0.001 | 75.00 ± 13.80 | 78.19 ± 14.24 | 0.228 |
| Previous PCI | 503 (9.6) | 488 (9.6) | 15 (6.8) | 0.142 | 486 (9.7) | 17 (6.9) | 0.133 |
| Numbers of diseased vessels |  |  |  | < 0.001 |  |  | 0.142 |
| 1 | 659 (12.6) | 636 (12.7) | 23 (10.4) |  | 627 (12.6) | 32 (12.9) |  |
| 2 | 1113 (21.2) | 1070 (21.3) | 43 (19.4) |  | 1057 (21.2) | 56 (22.6) |  |
| 3 | 2490 (47.5) | 2353 (46.9) | 137 (61.7) |  | 2364 (47.4) | 126 (50.8) |  |
| Killip |  |  |  | < 0.001 |  |  | 0.552 |
| 1 | 1340 (25.6) | 1294 (25.8) | 46 (20.7) |  | 1227 (24.6) | 113 (45.6) |  |
| 2 | 744 (14.2) | 694 (13.8) | 50 (22.5) |  | 709 (14.2) | 35 (14.1) |  |
| 3 | 368 (7.0) | 338 (6.7) | 30 (13.5) |  | 357 (7.2) | 11 (4.4) |  |
| 4 | 218 (4.2) | 190 (3.8) | 28 (12.6) |  | 199 (4.0) | 19 (7.7) |  |
| Diagnosis |  |  |  | < 0.001 |  |  | 0.709 |
| STEMI | 1232 (23.5) | 1150 (22.9) | 82 (36.9) | < 0.001 | 1097 (22.0) | 135 (54.4) | 0.709 |
| NSTE-ACS | 4008 (76.5) | 3958 (77.5) | 140 (63.1) |  | 3895 (78.0) | 113 (45.6) |  |
| Uric acid (μmol/L) | 352.64 ± 104.20 | 351.71 ± 103.69 | 373.51 ± 113.30 | 0.001 | 352.89 ± 103.62 | 347.63 ± 115.34 | 0.048 |
| Triglyceride (mmol/L | 1.84 ± 1.49 | 1.85 ± 1.48 | 1.82 ± 1.61 | 0.205 | 1.85 ± 1.50 | 1.72 ± 1.22 | 0.098 |
| Total cholesterol (mmol/L) | 4.40 ± 1.12 | 4.40 ± 1.12 | 4.43 ± 1.22 | 0.792 | 4.39 ± 1.12 | 4.48 ± 1.13 | 0.078 |
| Platelets (10^9^/L) | 206.06 ± 62.74 | 206.25 ± 62.58 | 201.77 ± 66.15 | 0.157 | 205.86 ± 63.04 | 210.03 ± 56.33 | 0.07 |
| Mean platelet volume (fL) | 10.69 ± 1.28 | 10.69 ± 1.28 | 10.64 ± 1.41 | 0.627 | 10.69 ± 1.28 | 10.65 ± 1.28 | 0.025 |
| Platelet distribution width (fL) | 15.46 ± 1.91 | 15.46 ± 1.91 | 15.51 ± 1.97 | 0.427 | 15.48 ± 1.91 | 15.20 ± 1.99 | 0.144 |
| Platelet large cell ratio (%) | 32.58 ± 7.78 | 32.58 ± 7.70 | 32.64 ± 9.47 | 0.872 | 32.61 ± 7.75 | 32.00 ± 8.38 | 0.076 |
| Lymphocyte (10^9^/L) | 1.58 ± 0.64 | 1.59 ± 0.64 | 1.36 ± 0.65 | < 0.001 | 1.59 ± 0.64 | 1.43 ± 0.63 | 0.25 |
| Neutrophil (10^9^/L) | 6.07 ± 3.26 | 6.00 ± 3.20 | 7.84 ± 4.14 | < 0.001 | 5.95 ± 3.18 | 8.53 ± 3.93 | 0.72 |
| Glucose (mmol/L) | 6.63 ± 2.79 | 6.58 ± 2.73 | 7.73 ± 3.71 | < 0.001 | 6.55 ± 2.70 | 8.26 ± 3.89 | 0.511 |
| HDL-C (mmol/L) | 1.10 ± 0.25 | 1.10 ± 0.25 | 1.09 ± 0.28 | 0.142 | 1.10 ± 0.25 | 1.10 ± 0.25 | 0.023 |
| LDL-C (mmol/L) | 2.36 ± 0.84 | 2.36 ± 0.84 | 2.37 ± 0.91 | 0.856 | 2.36 ± 0.84 | 2.45 ± 0.85 | 0.112 |
| Hemoglobin (g/L) | 132.27 ± 17.40 | 132.54 ± 17.23 | 126.00 ± 19.87 | < 0.001 | 132.32 ± 17.42 | 131.19 ± 16.98 | 0.066 |
| WBC (10^9^/L) | 8.31 ± 3.27 | 8.24 ± 3.20 | 9.90 ± 4.25 | < 0.001 | 8.19 ± 3.18 | 10.66 ± 4.06 | 0.677 |
| RBC (10^12^/L) | 4.33 ± 0.57 | 4.34 ± 0.56 | 4.14 ± 0.66 | < 0.001 | 4.33 ± 0.57 | 4.28 ± 0.56 | 0.093 |
| PLR (%) | 151.89 ± 84.89 | 150.64 ± 83.93 | 179.99 ± 100.27 | < 0.001 | 150.68 ± 84.13 | 176.19 ± 95.92 | 0.283 |
| NLR (%) | 4.87 ± 4.78 | 4.74 ± 4.61 | 7.65 ± 7.08 | < 0.001 | 4.74 ± 4.71 | 7.44 ± 5.37 | 0.536 |
| BNP (pg/mL) | 1789.99 ± 2409.87 | 1718.87 ± 2310.27 | 3397.60 ± 3714.14 | < 0.001 | 1762.77 ± 2390.41 | 2337.99 ± 2720.72 | 0.225 |
| Troponin (ng/Ml) | 3.53 ± 5.33 | 3.45 ± 5.31 | 5.19 ± 5.66 | < 0.001 | 3.29 ± 5.15 | 8.38 ± 6.50 | 0.869 |
| CKD | 207 (4.0) | 180 (3.6) | 27 (12.2) | < 0.001 | 194 (3.9) | 13 (5.2) | 0.065 |
| Hypertension | 3215 (61.4) | 3061 (61.0) | 154 (69.4) | 0.015 | 3079 (61.7) | 136 (54.8) | 0.139 |
| Hyperlipidemia | 662 (12.6) | 645 (12.9) | 17 (7.7) | 0.029 | 637 (12.8) | 25 (10.1) | 0.084 |
| Diabetes | 1885 (36.0) | 1801 (35.9) | 84 (37.8) | 0.603 | 1800 (36.1) | 85 (34.3) | 0.037 |
| ACEI | 3252 (62.1) | 3117 (62.1) | 135 (60.8) | 0.748 | 3104 (62.2) | 148 (59.7) | 0.051 |
| ARB | 1280 (24.4) | 1219 (24.3) | 61 (27.5) | 0.317 | 1235 (24.7) | 45 (18.1) | 0.161 |
| β-blockers | 4476 (85.4) | 4297 (85.6) | 179 (80.6) | 0.049 | 4264 (85.4) | 212 (85.5) | 0.002 |
| Diuretics | 1397 (26.7) | 1259 (25.1) | 138 (62.2) | < 0.001 | 1294 (25.9) | 103 (41.5) | 0.335 |
| CCB | 1683 (32.1) | 1611 (32.1) | 72 (32.4) | 0.977 | 1630 (32.7) | 53 (21.4) | 0.256 |
| Ticagrelor | 1478 (28.2) | 1381 (27.5) | 97 (43.7) | < 0.001 | 1342 (26.9) | 136 (54.8) | 0.593 |
| Morphine | 857 (16.4) | 775 (15.4) | 82 (36.9) | < 0.001 | 775 (15.5) | 82 (33.1) | 0.418 |
| Abbreviation: PCI, percutaneous coronary intervention; STEMI, ST-segment elevation myocardial infarction;NSTEMI-ACS, unstable angina/non-ST-segment elevation myocardial infarction;HDL, high density lipoprotein;LDL, Low density lipoprotein;WBC, white blood cell;RBC, red blood cell;PLR, platelet to lymphocyte ratio;NLR, neutrophil to lymphocyte ratio;BNP, brain natriuretic peptide;CKD, chronic kidney injury;ACEI, angiotensin converting enzyme inhibitors;ARB, angiotensin receptor blockers;CCB, calcium channel blocker. | | | | | | | |

**Table S2 Classification performance of eight machine learning models according to all-cause mortality within one year.**

| Classifier | AUC | Accuracy | Specificity | Recall (Sensitivity) | Precision | F1 score |
| --- | --- | --- | --- | --- | --- | --- |
| KNN | 0.68 | 0.80 | 0.41 | 0.81 | 0.98 | 0.87 |
| SVM | 0.74 | 0.92 | 0.20 | 0.94 | 0.98 | 0.94 |
| LDA | 0.83 | 0.85 | 0.68 | 0.85 | 0.99 | 0.90 |
| Decision Tree | 0.61 | 0.88 | 0.29 | 0.90 | 0.98 | 0.92 |
| XGBoost | 0.79 | 0.94 | 0.20 | 0.96 | 0.98 | 0.95 |
| RF | 0.81 | 0.88 | 0.56 | 0.89 | 0.99 | 0.92 |
| AdaBoost | 0.72 | 0.88 | 0.34 | 0.89 | 0.98 | 0.91 |
| CatBoost | 0.79 | 0.87 | 0.46 | 0.88 | 0.98 | 0.91 |
| Abbreviation: AUC,Area under the curve; KNN, K-nearest neighbor; SVM, Support vector machine; LDA, Linear discriminant analysis; RF, Random forest. | | | | | | |

**Table S3 Classification performance of the LDA model in all-cause mortality, AKI and MI.**

|  | AUC | Accuracy | Specificity | Sensitivity (Recall) | Precision | F1-score |
| --- | --- | --- | --- | --- | --- | --- |
| All-cause mortality | 0.83 | 0.85 | 0.68 | 0.85 | 0.99 | 0.90 |
| AKI | 0.74 | 0.80 | 0.56 | 0.82 | 0.97 | 0.86 |
| MI | 0.74 | 0.79 | 0.52 | 0.81 | 0.97 | 0.85 |
