# Supplementary figures and images for: Using machine learning to predict adverse events in acute coronary syndrome: A retrospective study
Source: Clin Cardiol. 2023 Aug 31;46(12):1594–602. doi: 10.1002/clc.24127 (PMC10716319; doi:10.1002/clc.24127)

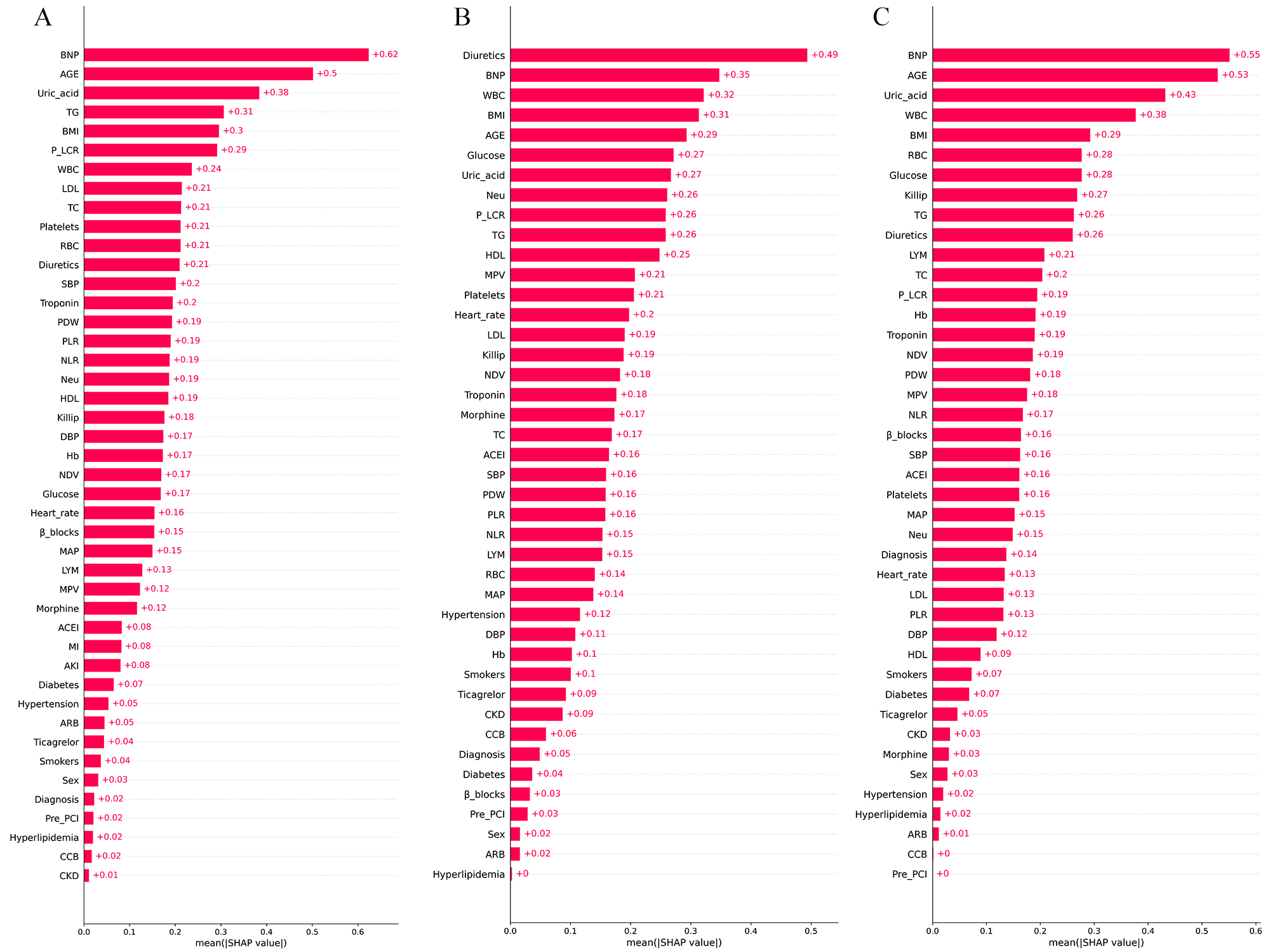

Supplement: Supplementary file 2 — Figure S1 Importance ranking of the candidate features. 1 A: Feature importance ranking for all‐cause mortality; 1B: Feature importance ranking for AKI;1 C: Feature importance ranking for MI. [file CLC-46-1594-s002.tif]
